# Recurrent Ventricular Tachycardia in a Young Adult—Imaging and Genetic Evidence of *DSP*‐Associated Arrhythmogenic Cardiomyopathy: A Case Report

**DOI:** 10.1155/cric/5899286

**Published:** 2026-04-07

**Authors:** Luis Enrique Gomez, Nicolas Martinenghi, Martin Ortiz-Genga, Mercedes Saenz Tejeira, Paula Buonfiglio, Andres Nicolas Atamañuk

**Affiliations:** ^1^ Department of Cardiology, Hospital de Agudos Juan A. Fernández, Buenos Aires, Argentina; ^2^ Health in Code, A Coruña, Spain

## Abstract

**Background:**

Ventricular tachycardia (VT) may represent the first manifestation of inherited cardiomyopathies, particularly in young patients without overt structural heart disease. Arrhythmogenic cardiomyopathy (ACM) is an inherited myocardial disorder characterized by ventricular arrhythmias, fibrofatty myocardial replacement, and an increased risk of sudden cardiac death. Pathogenic variants in the desmoplakin (DSP) gene have been increasingly associated with left‐dominant or biventricular forms of ACM and inflammatory “hot phases” of myocardial injury.

**Case Presentation:**

We report the case of a 37‐year‐old male presenting with sustained monomorphic VT with right ventricular outflow tract morphology requiring synchronized electrical cardioversion. Electrocardiography in sinus rhythm demonstrated low‐voltage limb leads and T‐wave inversion in V1–V3. Echocardiography showed mildly reduced right ventricular function with dyskinesia of the RV free wall (TAPSE 17 mm, RV S ^′^ 10 cm/s). Cardiac magnetic resonance revealed mild RV dilation and subepicardial late gadolinium enhancement in the lateral left ventricular wall with mild pericardial involvement, consistent with an ACM‐related inflammatory phenotype. An implantable cardioverter defibrillator was implanted for secondary prevention. Genetic testing identified a heterozygous pathogenic DSP frameshift variant (c.1009_1010dup; p.Leu338Serfs36∗), confirming the diagnosis of DSP‐related ACM.

**Conclusion:**

This case highlights the importance of integrating electrocardiography, multimodality imaging, and genetic testing in the evaluation of VT in young adults. Identification of a pathogenic DSP variant confirmed the diagnosis of ACM and has important implications for arrhythmic risk stratification and family screening.

Key Teaching Points

• *Recurrent ventricular tachycardia (VT) in young adults* warrants investigation for inherited cardiomyopathies beyond ischemic causes.
• *Electrocardiographic (ECG) patterns*, including T‐wave inversions in V1–V3 and low‐voltage limb leads. These findings should prompt consideration of arrhythmogenic right ventricular cardiomyopathy (ARVC) within the differential diagnosis.
• *DSP (desmoplakin) pathogenic variants* are a major cause of arrhythmogenic cardiomyopathy (ACM), often associated with left ventricular (LV) involvement, inflammatory myocardial injury, and high arrhythmic risk.
• *Genetic confirmation* provides diagnostic certainty and guides family counseling and risk stratification.

## 1. Introduction

VT is defined as a sustained ventricular arrhythmia originating in the ventricular myocardium, typically resulting in a heart rate exceeding 100 bpm [1]. VT may arise from re‐entrant circuits, triggered activity, or abnormal automaticity, in addition to its anatomical origin. It is a potentially lethal arrhythmia that may occur in both structurally normal and abnormal hearts and is a leading cause of sudden cardiac death [2]. The diagnosis of VT is usually made based on ECG data, most commonly a 12‐lead ECG.

The majority of VTs in structurally abnormal hearts are caused by myocardial fibrosis [3], which produces regions of electrical isolation and slow conduction that facilitate the re‐entry circuits. Similar to atrial flutter or fibrillation, this re‐entrant mechanism creates self‐sustaining activation loops that continue until they are interrupted [4]. A variety of diseases can cause fibrosis, classified as ischemic or nonischemic cardiomyopathies, with inflammatory etiologies considered within the nonischemic spectrum; these conditions are associated with characteristic scarring patterns and an increased risk of ventricular arrhythmias [5].

About 10% of cases in structurally normal hearts are caused by VT. The most common types are fascicular VTs that involve Purkinje re‐entry circuits in the LV and outflow tract tachycardias, which usually originate from the right ventricular outflow tract (RVOT) and can be detected by an ECG [6]. Although these arrhythmias are usually benign and well tolerated hemodynamically [7, 8], they can occasionally result in sudden cardiac death or cardiomyopathy caused by tachycardia [9]. Crucially, the existence of an outflow tract morphology should raise concerns about ACM, especially if it is accompanied by abnormal imaging or ECG results. In the present work, we use the term ACM to capture the broader spectrum of disease, including biventricular and left‐dominant forms, while acknowledging that current guidelines predominantly employ ARVC for diagnostic purposes.

## 2. Case Report

A 37‐year‐old male with a previous history of sustained VT in 2009 (treated with electrical cardioversion and normal coronary angiography) presented with abdominal pain, nausea, vomiting, and palpitations. The patient reported no family history of sudden cardiac death or known cardiomyopathy.

## 3. Examination

On arrival, his vital signs were as follows: blood pressure 118/60 mmHg, heart rate 180 bpm, and oxygen saturation 99%. He was alert, oriented, and well perfused.

## 4. ECG

**Figure 1:**
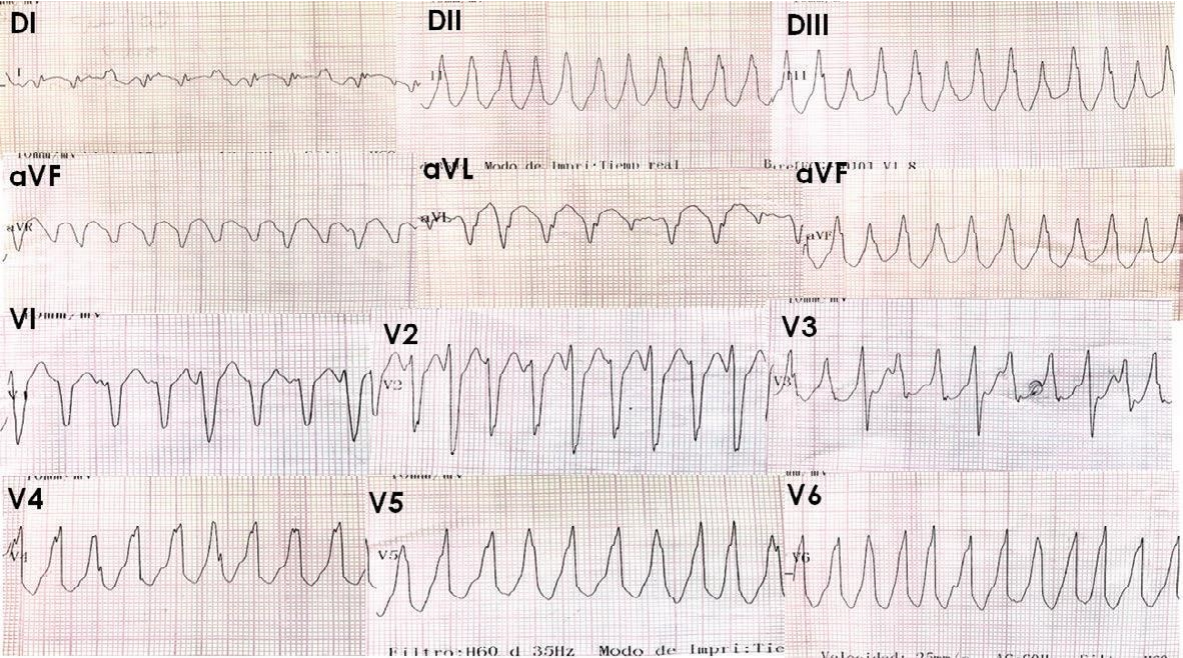
Twelve‐lead electrocardiogram showing monomorphic ventricular tachycardia with inferior axis and left bundle branch block (LBBB) morphology, consistent with an arrhythmia originating from the right ventricular outflow tract (RVOT).

The initial ECG (Figure 1) showed monomorphic VT with an inferior axis and left bundle branch block (LBBB) morphology, suggestive of an RVOT origin.

Because he was hemodynamically compensated, intravenous amiodarone was administered. However, due to subsequent hypotension, synchronized cardioversion (50 J) was performed, restoring sinus rhythm. The patient was subsequently admitted to the intensive care unit (ICU). Cardiac biomarkers were assessed at presentation. Creatine phosphokinase (CPK) was 45 U/L, while high‐sensitivity troponin was mildly elevated at 65.2 ng/L.

**Figure 2:**
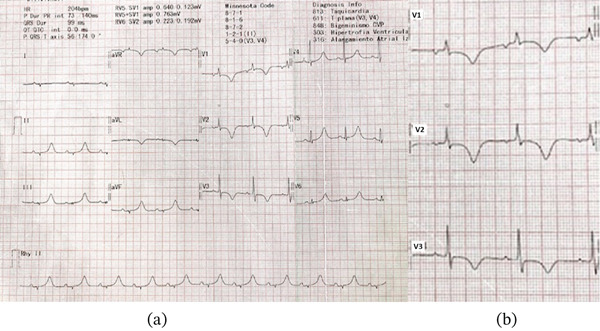
Electrocardiogram in sinus rhythm showing low‐voltage complexes in limb leads and (a) T‐wave inversion in V1–V3 with (b) an isoelectric J point, consistent with ACM.

A second ECG (Figure 2) in sinus rhythm showed low‐voltage QRS complexes in limb leads and T‐wave inversion in V1–V3 with an isoelectric J‐point, raising suspicion for ACM.

## 5. Imaging

Echocardiography demonstrated mildly reduced right ventricular (RV) systolic function with dyskinesia of the RV free wall. Quantitative assessment showed a tricuspid annular plane systolic excursion (TAPSE) of 17 mm and a tricuspid annular systolic velocity (RV S ^′^) of 10 cm/s, supporting mild RV systolic dysfunction. Coronary angiography was normal.

**Figure 3:**
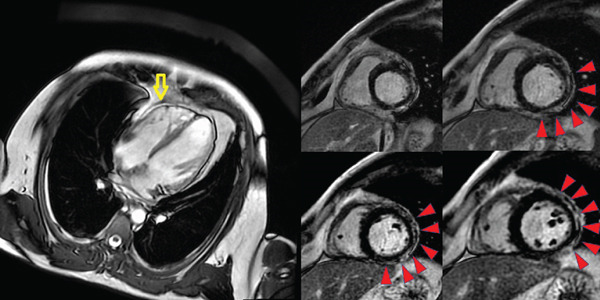
Cardiac magnetic resonance imaging. The yellow arrow indicates mild RV dilation and trabeculation with apical hypokinesia. The red arrow shows subepicardial LGE in the anterolateral and lateral LV segments, consistent with arrhythmogenic cardiomyopathy.

Cardiac magnetic resonance (CMR) revealed (Figure 3):

• Mild RV dilation and trabeculation with apical hypokinesia
• LV with preserved diameters and mildly reduced ejection fraction (46%), with trabeculation in apical segments and mild regional hypokinesia
• Subepicardial late gadolinium enhancement (LGE) in anterolateral and lateral LV segments, with associated pericardial thickening and mild pericardial effusion
• No fatty infiltration in the RV free wall

Although mild subepicardial LGE and pericardial involvement were present, the findings were interpreted mainly as an ACM‐related inflammatory phenotype.

During hospitalization in the ICU, given the recurrence of sustained VT, an implantable cardioverter defibrillator (ICD) was placed for secondary prevention of sudden cardiac death. The procedure was uneventful, and device interrogation confirmed appropriate sensing and capture parameters. The patient was discharged in stable condition with no further arrhythmic episodes during follow‐up.

## 6. Genetic Testing

Based on the phenotypic findings, we performed targeted genetic testing, which revealed a heterozygous pathogenic frameshift variant in the *DSP* gene (NM_004415.4: c.1009_1010dup; p.Leu338Serfs36∗), resulting in premature protein truncation and functional haploinsufficiency. The variant was classified as pathogenic according to the American College of Medical Genetics and Genomics and the Association for Molecular Pathology (ACMG/AMP) guidelines. This finding confirmed the diagnosis of DSP‐related ACM. Cascade genetic screening was advised for first‐degree relatives.

## 7. Discussion

Ventricular arrhythmias, fibrofatty replacement of the ventricular myocardium, and the possibility of sudden cardiac death are the hallmarks of ACM, an inherited myocardial condition [10, 11]. Among these, disease‐causing variants in *DSP* have been increasingly recognized and are frequently associated with LV involvement and inflammatory “hot phases” of myocardial injury [12, 13].

We report a case of recurrent VT in a young adult whose genetic testing for a pathogenic *DSP* variant confirmed that the patient had ACM phenotypic features.

The patient had imaging evidence of both RV and LV involvement, recurrent VT, and ECG abnormalities suggestive of ACM, consistent with the 2020 Padua criteria, which incorporate ventricular morphofunctional abnormalities [14]. Subepicardial LGE and pericardial enhancement were detected by CMR, which is in line with the “inflammatory hot phase” phenotype commonly seen in cardiomyopathy linked to *DSP*.

DSP is a critical desmosomal protein that anchors intermediate filaments to the desmosomal structure. Loss‐of‐function variants, such as the frameshift variant identified in this case (*DSP* c.1009_1010dup; p.Leu338Serfs36∗), have been associated with ACM, often with left‐dominant involvement and an increased risk of ventricular arrhythmias, although individual phenotypic expression may vary. This truncating variant is located within the constitutive nonsense‐mediated decay (NMD)–competent region in the globular head of the gene.

Recent studies suggest that variant location may represent a risk factor for arrhythmic events, providing valuable insights that can guide variant interpretation for both variant interpretation and precision‐based clinical management. Specifically, *DSP* variations found in the central rod and globular head domains have been linked to higher arrhythmic burden, earlier onset, and more aggressive disease phenotype in some cases, though variability among patients is observed [15].

Genetic confirmation is crucial not only for diagnosis but also for risk stratification and family screening.

## 8. Conclusions

Recurrent VT in young adults should prompt consideration of inherited cardiomyopathies. This case underscores the importance of combining ECG, imaging, and genetic testing. Identification of a pathogenic *DSP* variant confirmed the diagnosis of ACM and has significant implications for arrhythmic management and family evaluation.

## Funding

No funding was received for this manuscript.

## Consent

Written informed consent was obtained from the patient for the publication of this case report and any accompanying images.

## Conflicts of Interest

The authors declare no conflicts of interest.

## Data Availability

Data sharing is not applicable to this article as no datasets were generated or analyzed during the current study.
